# Comparative Analysis of PacBio and Oxford Nanopore Sequencing Technologies for Transcriptomic Landscape Identification of *Penaeus monodon*

**DOI:** 10.3390/life11080862

**Published:** 2021-08-23

**Authors:** Zulema Udaondo, Kanchana Sittikankaew, Tanaporn Uengwetwanit, Thidathip Wongsurawat, Chutima Sonthirod, Piroon Jenjaroenpun, Wirulda Pootakham, Nitsara Karoonuthaisiri, Intawat Nookaew

**Affiliations:** 1 Department of Biomedical Informatics, University of Arkansas for Medical Sciences, Little Rock, AR 72205, USA; zdominguez@uams.edu (Z.U.); twongsurawat@uams.edu (T.W.); pjenjaroenpun@uams.edu (P.J.); 2 National Center for Genetic Engineering and Biotechnology (BIOTEC), National Science and Technology Development Agency, Pathum Thani 12120, Thailand; kanchana.sit@biotec.or.th (K.S.); tanaporn.uen@biotec.or.th (T.U.); nitsara.kar@biotec.or.th (N.K.); 3 Division of Bioinformatics and Data Management for Research, Department of Research and Development, Faculty of Medicine, Siriraj Hospital, Mahidol University, Bangkok 10700, Thailand; 4 National Omics Center (NOC), National Science and Technology Development Agency (NSTDA), Pathum Thani 12120, Thailand; chutima.son@nstda.or.th (C.S.); wirulda.poo@nstda.or.th (W.P.)

**Keywords:** long read sequencing, transcriptomics, Oxford Nanopore Technologies, PacBio, *Penaeus monodon*

## Abstract

With the advantages that long-read sequencing platforms such as Pacific Biosciences (Menlo Park, CA, USA) (PacBio) and Oxford Nanopore Technologies (Oxford, UK) (ONT) can offer, various research fields such as genomics and transcriptomics can exploit their benefits. Selecting an appropriate sequencing platform is undoubtedly crucial for the success of the research outcome, thus there is a need to compare these long-read sequencing platforms and evaluate them for specific research questions. This study aims to compare the performance of PacBio and ONT platforms for transcriptomic analysis by utilizing transcriptome data from three different tissues (hepatopancreas, intestine, and gonads) of the juvenile black tiger shrimp, *Penaeus monodon*. We compared three important features: (i) main characteristics of the sequencing libraries and their alignment with the reference genome, (ii) transcript assembly features and isoform identification, and (iii) correlation of the quantification of gene expression levels for both platforms. Our analyses suggest that read-length bias and differences in sequencing throughput are highly influential factors when using long reads in transcriptome studies. These comparisons can provide a guideline when designing a transcriptome study utilizing these two long-read sequencing technologies.

## 1. Introduction

The advent of single-molecule, third-generation sequencing technologies, mainly represented by Pacific Biosciences (PacBio) and Oxford Nanopore Technologies (ONT), marked the beginning of a new era in genomics [1]. Thus, many other “-omics” are quickly adapting to these technologies, further improving the accumulated scientific knowledge obtained during the past decades using short-read sequencing methods. Although Illumina (short read) is still a widely used sequencing platform for transcriptomic studies, it has some technological limitations that can be overcome using long-read sequencing [2,3,4]. The main limitations of short-read sequencing are that assembled transcripts from short reads do not cover full-length transcripts in eukaryotic genomes, and PCR amplification bias can be introduced during library construction [5,6]. Read lengths are over 15 kb for PacBio and 30 kb for ONT, exceeding the length needed to cover most RNA molecules in eukaryotes [2]. Additionally, sample preparation for ONT long-read sequencing does not require PCR amplification, thus reducing possible bias, although with the consequence of reduced sequencing throughput [1,2,7]. Moreover, previous work comparing Illumina and ONT quantification of gene expression assessed the existence of a high level of correlation between the quantification of transcripts for both technologies [8,9,10,11], confirming that results obtained with ONT RNA-seq approaches are comparable to those obtained with short-read sequencing platforms. All of these characteristics make these two long-read sequencing technologies—ONT and PacBio—very attractive and suitable options for isoform and fusion transcript discovery and de novo transcriptomic analysis [12].

While encouraging, third-generation sequencing platforms are still improving, and analysis of long-read transcriptomes is still challenging. One of the main drawbacks of leveraging third-generation sequencing technologies is the relative lack of bioinformatic tools and standardized pipelines designed for downstream analysis [13]. In the same vein, the raw base-called error rate, although improved in recent years, is still reported to be <1% in circular consensus reads (CSS) from PacBio [14] and <5% for ONT sequences [15]. Characteristics of the error distribution also vary between both technologies [6,16], with mainly randomly distributed indels for PacBio reads and indels in homopolymer regions for ONT reads [16]. Nonetheless, recent improvements in the sequencing throughput of both third-generation sequencing technologies have made it possible to conduct the first studies of full eukaryotic transcriptomes using only long-read sequencing platforms without Illumina sequencing [13]. However, there is still a lack of benchmarking studies and information about the suitability of applications for long-read sequencing technologies, especially when applied to transcriptomic studies in eukaryotes and transcriptional landscape analysis.

In this study, we aimed to compare PacBio and ONT reads by investigating the transcriptomic landscape of an economically important shrimp species, the black tiger shrimp, *Penaeus monodon*. With an origin in the Indo-West Pacific waters, this shrimp species is one of the dominant cultured species worldwide, accounting for 9% of total crustacean production [17]. Characteristic of decapod crustaceans, the *P. monodon* genome is large and highly repetitive, making long-read sequencing techniques very well suited for genomic characterization. The first genome assembly of *P. monodon* was carried out in 2016 using Illumina paired-end reads [18], resulting in a highly fragmented draft genome with over 2 million scaffolds and less than 35% completeness [18]. An improved *P. monodon* genome assembly was released in 2019 [19], leveraging a hybrid approach using long reads from ONT MinION sequencing and short reads from paired-end Illumina sequencing. Recently, a high-quality chromosome-level *P. monodon* genome was achieved by our team using a combination of PacBio high-depth sequencing and Illumina sequencing, with the final scaffolding performed using long-range Chicago and Hi-C technologies [20].

**Figure 1:**
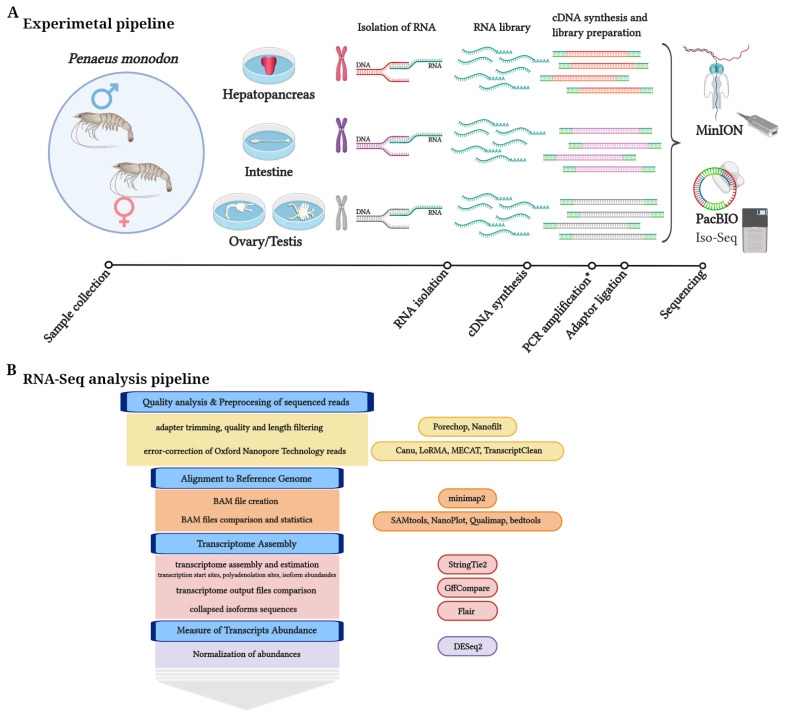
(**A**) Experimental and (**B**) bioinformatic pipelines used in this study. Asterisk (*) indicates a step in the pipeline required for PacBio sequencing. Figure was made using BioRender.com.

In this study, we took advantage of the recent high-quality *P. monodon* assembly, which is a prerequisite for transcriptomic landscape analysis, to assess the performance of PacBio and ONT reads; our goal was to evaluate and compare both technologies in each of the different stages of a general transcriptomic pipeline using samples from three different tissues from male and female *P. monodon* (Figure 1). The library generated by PacBio rendered 542,686 high-quality circular consensus sequence (CCS) reads with an average length of 2764 base pairs (bp), while the library generated by ONT rendered 34,202,609 filtered reads with an average length of 1216 bp. First, we compared the main characteristics of the two sequencing libraries. Second, we used StringTie2 [21] to compare the different profiles of assembled transcripts for both technologies, using their assembled intron chains and comparing them with those from the transcripts annotated in the reference genome. Third, we determined the degree of correlation of the level of gene expression between all samples of the study. Finally, we assessed the level of completeness of the final assembled transcriptomes using the Benchmarking Universal Single-Copy Orthologs (BUSCO) genes from 90 arthropod species.

Our findings highlight that read-length bias and differences in sequencing throughput between both technologies are key aspects to consider when designing transcriptomic studies that use these long-read sequencing technologies.

## 2. Materials and Methods

### 2.1. Sample Collection and RNA Preparation

Two four-month-old (male and female) juvenile *P. monodon* (black tiger shrimp) were collected from the Prachuapkhirikhan province in Thailand. The hepatopancreas, intestine, and gonads (testes and ovaries) were dissected and immediately frozen in liquid nitrogen and stored at −80 °C before RNA extraction. Total RNA was extracted from 50 mg frozen tissues using a TRI Reagent method according to the manufacturer’s instructions (Molecular Research Center, Cincinnati, OH, USA). Genomic DNA was eliminated using 0.5 U/μg of RNase-Free DNase I (Promega, Madison, WI, USA) at 37 °C for 30 min. The purified RNA was then aliquoted for PacBio and ONT sequencing.

### 2.2. PacBio Library Preparation and Sequencing

Total RNA (1 µg per sample) from the male and female *P. monodon* hepatopancreas, intestine, and gonads was sequenced using the IsoSeq method (NovogeneAIT, Singapore). The RNA quality measurements are provided in Supplementary Table S1. Six libraries were constructed using the SMRTbell Template Prep Kit 2.0 (Pacific Bioscience, Menlo Park, CA, USA) and NEBNext single cell/low input RNA library (New England BioLabs, Ipswich, MA, USA). Each library was constructed for each sample, and they were run on PacBio Sequel to generate minimum output of 4G per sample. The sequencing was performed on a Sequel sequencing kit 3.0, SMRT cell 1 M v3 LR. SMRTlink software v7.0 was used to filter and process the raw sequencing subreads with the cutoff or read quality ≥ 0.8 126 (minReadScore = 0.8). SMRTlink software v7.0 was used to filter and process the raw sequencing subreads with the cutoff or read quality ≥ 0.8 (minReadScore = 0.8). Filtered subreads were processed using the IsoSeq3 pipeline to obtain highly accurate long reads. The IsoSeq3 pipeline included three main steps. First, the subreads were clustered based on circular consensus sequences (CCS) using the “ccs” module from SMRTlink v7.0 software with the following parameters: at least a single polymerase on a single strand of an insert within a SMRTbell™ template and no adapter sequences (minPasses = 1) and minimum predicted accuracy of 0.9 (minPredictedAccuracy = 0.9). The CCS produced HiFi (high fidelity) reads that could improve > 99% single-molecule read accuracy. Second, the HiFi reads were defined as full-length non-chimeric (FLNC) or non-full-length, depending on the presence or absence of both 5′ and 3′ primers at the read ends. The “lima” and IsoSeq3 “refine” modules in SMRTlink were applied for this step. The “lima” identified and removed the 5′ and 3′ cDNA primers, while IsoSeq3 “refine” removed polyA tails and artificial concatemers to produce refined CCS reads. Finally, FLNC reads were clustered into isoforms using the IsoSeq3 “cluster” module in SMRTlink. The clusters were polished using IsoSeq3 polish, and a consensus sequence was created for each clustered transcript. All six final sequencing read libraries are available in GenBank under project ID PRJNA602748.

### 2.3. ONT Library Preparation and Sequencing

The ONT Direct cDNA 1D Kit (SQK-DC109) was used for PCR-free cDNA library preparation according to the ONT-recommended protocol for each sample (male and female *P. monodon* hepatopancreas, intestine, and gonads). To avoid RNA shearing, total RNA (4000 ng) was used for the input material for library preparation without purification for mRNA. Each prepared library was loaded onto a MinION flow cell (R9.4.1 version/FLO-MIN106) with MinKNOW software version 1.7.14 (ONT) for a sequencing run lasting 48 h to generate fast5 files. The fast5 files were converted to base-called .fastq files using the base-caller Guppy version 2.3.4 software. ONT reads were pre-processed with Porechop v0.2.3 (https://github.com/rrwick/Porechop, accessed on 1 August 2020). They were also filtered to be longer than 200 nt using NanoFilt from NanoPack [22]. Read quality was calculated with NanoStat v1.2.0 from NanoPack [22]. All six final sequencing read libraries are available on GenBank database under project ID PRJNA602748.

### 2.4. ONT Self-Correction of Reads

To overcome the sequencing accuracy limitations of ONT MinION, four algorithms were used for error-correction of ONT reads. Canu v2.0 [23] and LoRMA [24] did not produce results for our dataset after 1 month of execution using a compute node with 36 2.1 GHz-Xeon cores and 128 GB of RAM, with parameters “--correct stopOnReadQuality = false stopAfter = readCorrection” for Canu V2.0 and default parameters for LoRMA v0.5 due to the high-throughput ONT reads. However, these software have been used successfully for this purpose with smaller datasets [25].

TranscriptClean v2.0.2 [26] was used with “basic options” with SAM file format of aligned reads as an input. MECAT v1.0 [27] software was used with options “-j 0 -x 1 -n 50 -a 100” for *mecat2pw* and options “-I 0 -x 1 -a 100 -c 1 -l 200” for *mecat2cns*.

### 2.5. Transcriptome Analyses: Assembly of High-Quality Long-Reads Using StringTie2

Transcriptomic analyses were performed using the 1D-trimmed and -filtered ONT mapped reads, as well as mapped products from TranscriptClean v2.02 along with the refined CCS mapped reads from the IsoSeq3 pipeline. ONT and PacBio reads were aligned against the reference genome using minimap2 [28] in spliced alignment mode. The resulting SAM files were converted into BAM files and then sorted and indexed with SAMtools v1.9. Comparison of the resulting set of BAM files and further statistical analyses of mapped reads and BAM files were performed using SAMtools v1.9 [29,30], NanoStat v1.2.0 [22], Nanoplot v1.29.0 [22], QualiMap v2.2.2 [31], BedTools v2.29.2 [30], and BEDOPS v2.4.39 [32]. BAM files from ONT and PacBio samples were used as input for StringTie2 v2.1.4 [21] to generate expression estimates with parameters -L -A -C -B -G. Resulting general transfer format (GTF) files with assembled transcripts from the first round were used with “merge” mode (--merge). The merge mode of StringTie2 merges the transcripts annotated in the GTF files from multiple experiments, into a non-redundant, unified set of transcripts. Thus, this option creates a consensus annotation of all transcripts (from the reference provided annotation and de novo). Final transcripts were obtained after filtering the transcript length by >200 nt. Junctions were supported by a coverage of at least 1. Assembled transcripts from StringTie2 using the merged GTF file were used with GffCompare from GFF utilities v0.11.5 (https://ccb.jhu.edu/software/StringTie/gff.shtml, accessed on 1 August 2020) provided by Cufflinks [33] to compare the outputs with the original annotation file and to estimate the accuracy of all the GTF files obtained after StringTie2 assembly. A python script provided with StringTie2 (prepDE.py) was used to generate the gene and transcript count matrices from StringTie2 coverage values [34]. Final isoform sequences for transcripts from ONT samples were obtained with FLAIR v1.5.1 [35].

### 2.6. Correlation Analysis of Gene Expression Level

Mapped reads were quantified for each transcript and gene using the python script provided with StringTie2 (prepDE.py), generating transcripts and gene matrices of counts for ONT and PacBio data [34]. As previously reported, the negative binomial statistic is a proper method for gene expression analysis of long-read data [1]. Therefore, raw counts were normalized using DESeq2 [36] in R [37]. Spearman’s correlation coefficient was used to evaluate the correlation between normalized read values by tissue using the *cor* function from R [37] and by genes using a python script with *corrwith* function from *pandas*.

### 2.7. Assessment of Final Transcriptomes for Completeness and Functional Assignment

Completeness of isoforms obtained for both technologies was assessed with BUSCO v.4 [38] using the arthropoda_odb10 database [39]. The final isoforms were functionally annotated using their nucleotide sequence with the KEGG database using the online KEGG Automatic Annotation Server (http://www.genome.jp/tools/kaas/, accessed on 1 August 2020) with bi-directional best hit (BBH) method [40] and UniprotKB database (https://www.uniprot.org/, accessed on 1 August 2020) [41].

## 3. Results and Discussion

### 3.1. Assessment of Characteristics of PacBio SMRT and ONT Read Libraries

To compare the performance of Pacific Biosciences (PacBio) and Oxford Nanopore Technologies (ONT) methodologies, we sequenced cDNA libraries from three different tissues (hepatopancreas, intestine, and gonads) from male and female *Penaeus monodon* (black tiger shrimp) using the PacBio Sequel System and ONT MinION sequencer. These tissues were chosen because they are metabolically active tissues in both sexes that will increase the proxy of number expressed genes obtained from the genome. The experimental and RNA-seq analysis pipelines are presented in Figure 1. Unlike the PacBio approach, ONT reads were produced by direct cDNA sequencing without PCR amplification. A summary of the sequencing throughput for both technologies is shown in Table 1, and detailed statistics on the final reads used in the study are provided in Supplementary Table S1. Note that numbers for PacBio libraries in Table 1 correspond to “subreads.” In PacBio IsoSeq3 technology, each polymerase read is partitioned to form one or more subreads. These contain the sequences from a single pass of the polymerase on a single strand of an insert within a SMRTbell™ template without adapter sequences.

**Figure 2:**
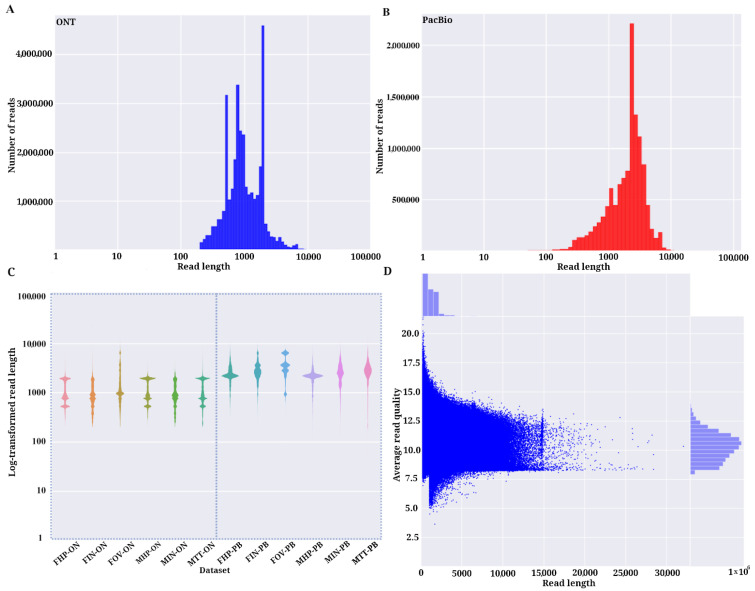
Comparison of ONT and PacBio sequencing libraries. (**A**) Bar plot of the number of ONT reads and read length after nanopore sequencing. (**B**) Bar plot of the number of PacBio subreads and read length. (**C**) Violin plot of the base 10 logarithmic transformation of the read length of the filtered reads for ONT samples and refined CCS reads for PacBio samples. (**D**) Average of read quality and read length of filtered ONT reads. Plots generated with Nanopack package [22].

As observed in Table 1, the sequencing yield for ONT libraries was much higher than for PacBio libraries. However, the full-length cDNA libraries generated for each sample had different sizes, ranging from 5.5 to 14 sequenced Gb for ONT and 3.5 to 5 sequenced Gb for PacBio (Table 1, Supplementary Figure S1A). The overall read lengths of libraries differ for both technologies (Supplementary Figure S1B). While the longest reads of the dataset belonged to ONT libraries (>27 kb in all 6 samples), the highest proportion of longer long-reads was found in PacBio libraries (>2.2 kb mean read length). Thus, mean read lengths from CCS PacBio libraries were in all cases longer than those from ONT, with more than ~1 kb difference (Supplementary Table S1). Figure 2A shows an irregular distribution of the read lengths from ONT libraries with many read length peaks at different sizes and the main read length distribution skewed to the left when compared to the PacBio read length distribution, which appears to have a more normalized shape (Figure 2B). Lower values in mean read length in ONT libraries might have been due to the presence of a higher proportion of fragmented reads that could be the sequencing product of non–full-length transcripts. This could have been caused by RNA degradation during library preparation protocols or software artifacts during the base-calling process [21,42].

After polyA tail trimming and concatemer removal steps using the IsoSeq3 pipeline, an average of 0.1 Mb full-length non-chimeric (FLNC) CCS reads were obtained from PacBio libraries (Supplementary Table S1). Once ONT reads were trimmed and filtered, ONT libraries contained more than 7 Gb on average, which is within the normal range for direct cDNA sequencing libraries according to ONT cDNA sequencing guidelines (https://nanoporetech.com, accessed on 1 August 2020). The distribution of final read lengths for both platforms is shown in Figure 2C. The PacBio IsoSeq3 pipeline produces highly accurate consensus reads by definition (>99% accuracy, Q > 20, https://www.pacb.com/smrt-science/smrt-sequencing/, accessed on 1 August 2020), but quality measurements of the CCS reads are not provided. Analysis of the average read quality of ONT 1D reads showed that most of the filtered sequencing products had mean quality scores (Q-scores) ranging from 9 to 12.5 (Figure 2D).

### 3.2. Analysis of Aligned Reads

**Figure 3:**
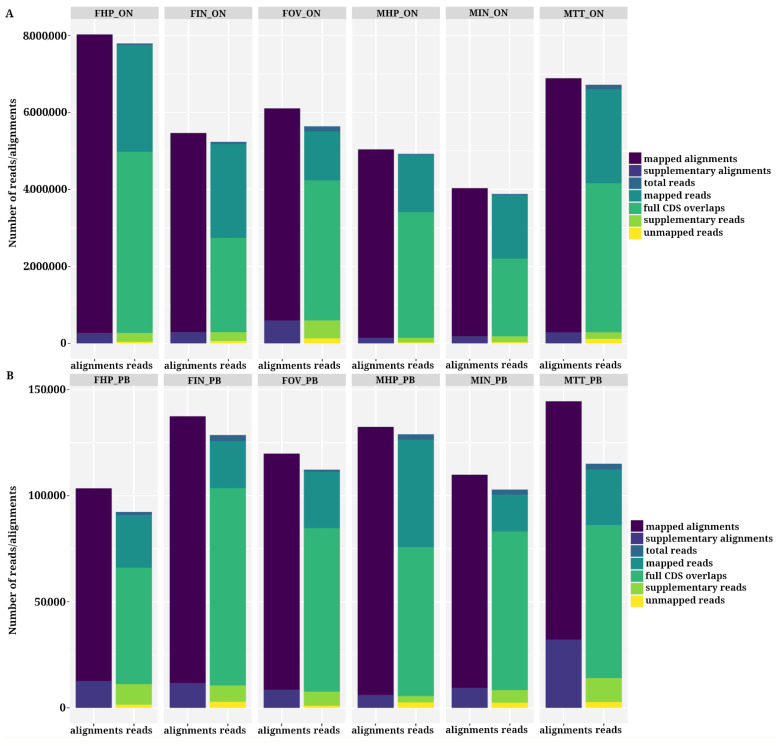
Analysis of the aligned reads against the reference file using minimap2. Bar plots of aligned reads (1st bar) and of total reads (2nd bar) using different classifications according to samtools [29] and Qualimap2 [31] statistics, for ONT samples (**A**) and PacBio samples (**B**).

The recently published chromosome-level *P. monodon* assembly [20] was used as the reference genome for read alignment using minimap2 [28]. The alignment rates were 98.8% for the ONT reads and 98.5% for the PacBio reads (Supplementary Table S3), while the average percent identity of the aligned reads to the reference, as calculated by NanoStat [22], was 90.1% for ONT and 94.5% for PacBio. The general alignment error rates, calculated as the sum of the number of mismatches (given by the NM auxiliary tag) divided by the number of aligned bases [31], were <0.12 for ONT and <0.06 for PacBio samples (Supplementary Table S3). Analysis of the distribution of the percent identity of the reads and their lengths when aligned to the reference genome for both technologies showed that the distribution of refined PacBio CCS reads was densest around values >90% percent identity, reflecting the lower error rate of the refined CCS reads when compared to the ONT reads, which presented a more relaxed distribution along the Y axis (Supplementary Figure S2). When analyzing the read alignments, >70% of the ONT reads and >75% of the PacBio reads overlapped with full-length coding sequences according to the annotation of the reference genome (Figure 3). Despite the higher number of ONT (Figure 3A) alignments relative to PacBio (Figure 3B) CCS mapped alignments (~60 fold greater for ONT) and the slightly higher error rate of the ONT reads, the percentage of unmapped reads in ONT samples (1.2% on average) was smaller than that of PacBio (1.5% on average) in almost all cases (Figure 3B and Supplementary Table S3).

Supplementary alignments are those from reads that cannot be mapped in a linear fashion; therefore, they are aligned to multiple parts of the reference genome [43]. Such alignments are also called chimeric alignments. Chimeric transcripts can be indicative of structural variations and genomic rearrangements in transcriptomic studies [44]. However, chimeras can also be due to technical artifacts from the reverse transcriptase polymerase chain reaction (RT-PCR) or failure to remove read adapters [44,45]. In our dataset, ONT samples presented a higher proportion of supplementary reads (4.89% on average) than PacBio. However, the number of alignments classified as supplementary was higher for PacBio samples (7.28% on average for each sample), while ONT supplementary alignments had a mean value < 5%.

### 3.3. Error Correction for ONT Reads

Due to possible differences in quality between ONT and PacBio reads (Supplementary Figure S2), we evaluated the outputs from two DNA self-correction tools for ONT reads: TranscriptClean v2.0.2 [26], and MECAT v1.0 [27]. The selection of these tools was based on results of previous benchmarking studies in which the effect of correction on detection of gene families, isoform diversity, bias toward the major isoforms, and splice site detection was evaluated [24,25]. The performance of these two error-correction programs is shown in Table 2. Due to the high throughput of the ONT reads, error correction steps from Canu and LoRMA software did not produce results for our dataset after 1 month of execution (using different configurations on a compute node with 36 2.1GHz-Xeon cores and 128 GB of RAM). On the other hand, TranscriptClean [26] and MECAT [27] were able to generate the corrected output (Table 2).

TranscriptClean corrects mismatches, microindels, and non-canonical splice junctions on reads already aligned to the reference genome [26]. The average read length and the general error rate (as provided by samtools stats v1.9) were improved in all samples when using both error-correction algorithms. In the case of MECAT, although computationally efficient, this software is oriented to produce reference-quality assemblies [27], and it works by overlapping candidate reads to reduce their “noise.” Therefore, although the quality of the reads was improved, the coverage was greatly reduced, making its output less desirable for quantification studies (Table 2).

### 3.4. Transcript Assembly and Identification of New Transcripts Using StringTie2

Although reads produced by third-generation sequencing methods are, in theory, long enough to cover whole transcripts, there are some scenarios where recovery of entire transcripts is not possible: quick degradation of RNA molecules before sequencing; long-read molecule rupture during library preparation; and failure of the reverse transcription step in cDNA sequencing [21,42]. To address these issues, we used StringTie2, which is a transcriptome assembler that can estimate transcript abundance based on the number of long reads mapped to each transcript. StringTie2 also offers a reference-free assembly method that allows detection of novel genes and isoforms that are not covered by the reference annotation file [21].

The number of genes identified by StringTie2 in the *P. monodon* genome was 32,664, which corresponds to 60,594 transcripts. This is a difference of 1114 genes and 24,218 transcripts with respect to the original genome annotation [20]. Although these numbers seem strikingly high, they are within the range of other studies using eukaryotic genomes that leveraged long-read sequencing to improve the genome annotation using transcriptomic analysis [13,46,47]. Despite the refinement of the transcript sequences, comparison of the individual merged transcript files obtained using ONT and ONT-corrected reads from TranscriptClean showed that the number of genes and transcripts annotated by StringTie2 were exactly the same. However, TranscriptClean is able to correct microindels and non-canonical splice junctions apart from mismatches of the aligned reads. Thus, assembled transcripts generated by StringTie2 were further analyzed to find structural changes in the identified transcripts, as against the reference annotation.

GffCompare [33] utility provides “class codes” (detailed in https://ccb.jhu.edu/software/StringTie/gffcompare.shtml, accessed on 1 August 2020, and in Supplementary Table S4) for each of the assembled transcripts annotated by StringTie2, as well as various statistics related to the accuracy of the input transcripts when compared to a reference annotation file. Table 3 shows measurements of sensitivity and precision, calculated at three levels: base, intron chain, and transcript. In short, measurements of sensitivity and precision were calculated according to the presence/absence of each of the features (bases in exons, intron chain, and transcripts: single and multi-exon) in each sample and in the reference annotation file. Statistical analyses were performed using two reference files. First, the original reference annotation file for *P. monodon* (GenBank: GCA_015228065.1) and later, the final merged file obtained with the -merge option of StringTie2, which is a global GTF file that contains the original set of annotated transcripts with a non-redundant set of novel transcripts annotated by StringTie2 after its first execution on our samples.

As observed in Table 3, all measurements of sensitivity and precision were improved when using the merged file with the novel annotated transcripts as a reference, indicating that transcripts annotated in the merged reference file matched with higher fidelity to the aligned transcripts from the bam files for all samples. Moreover, both technologies identified transcripts not previously annotated without significant variation in the estimated accuracy when evaluating the correctness of the set of predicted transcripts against the reference file. However, not all samples contributed the same number of novel transcripts to the final GTF annotation file.

**Figure 4:**
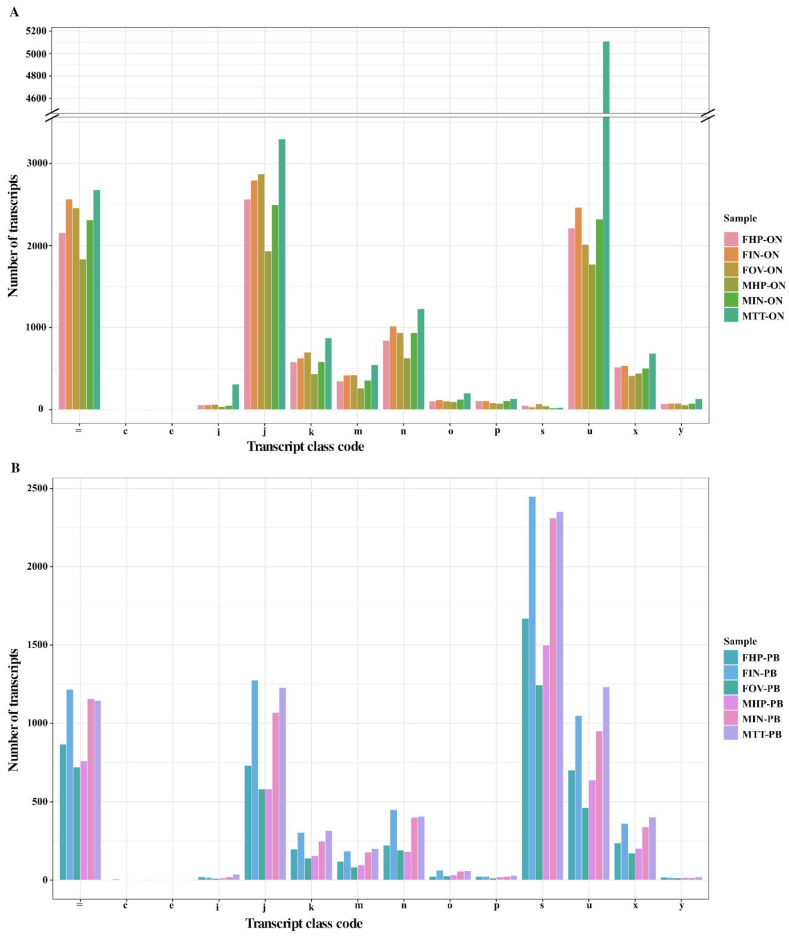
Analysis of transcript class codes. Analysis of transcripts obtained after alignment using StringTie2 and compared with the reference file. Annotation codes correspond to transcript classification codes obtained using the transcript file output from StringTie2 for each ONT sample (**A**) and PacBIO samples (**B**) and the reference file, and compared using GffCompare [33]. It should be noted that values obtained for ONT filtered samples and ONT samples after error correction with TranscriptClean were identical; therefore, they are not shown here. Code letters on the horizontal axis are further explained in Supplementary Table S4.

Results from the comparison of the transcripts assembled by StringTie2 to the annotated transcripts of the original reference file are presented in Figure 4 and in Supplementary Table S4. Transcripts were classified in different classes according to how their intron chains are mapped to the reference genome file using GffCompare (https://ccb.jhu.edu/software/stringtie/gffcompare.shtml, accessed on 1 August 2020). A high number of the annotated transcripts (25% of ONT and 16% of PacBio transcripts) belonged to the class code *j*, which corresponds to multi-exon transcripts in which at least one junction matches the annotation from the reference file (Figure 4). These are transcripts that do not fully match the reference but share at least one exon–intron junction. The transcripts annotated with the symbol “=” (22% of ONT and 17% of PacBio transcripts) correspond to those with intron chains that completely match the annotation in the reference file (a variation of 100 bases in the coordinates of the first and final exons was allowed). Interestingly, a high number of transcripts (34%) from PacBio samples (Figure 4B) were classified as class code *s*, which corresponds to transcripts in which the intron matched the opposite strand from the reference file. Further comparisons using the final output of IsoSeq3 pipeline (after “cluster” step), showed that this mapping errors observed in PacBio transcripts, are due to the presence of misoriented reads as a consequence of using refined CCS reads, which are not the final product of the IsoSeq3 pipeline (Supplementary Figure S3). Class *m* (4% of ONT and 3% of PacBio transcripts) and *n* (9% of ONT and 5% of PacBio transcripts) transcripts correspond to transcripts with retained introns, while class code *k* represents longer chains of transcripts that contain the reference (6% of ONT and 4% of PacBio transcripts). Transcripts classified as *u* (25% of ONT and 14% of PacBio transcripts) represent putative new transcripts that were not annotated in the reference file. This category would represent all novel transcripts identified by StringTie2 in the first round of assembly. As can be seen in Figure 4, most of the annotated unknown transcripts came from ONT sample MTT-ON, with more than 5000 of these novel (redundant) transcripts found in the MTT-ON testis sample (34% of all MTT-ON annotated transcripts). It is worth noting that sample MTT-PB also harbored the highest number of newly identified transcripts in PacBio samples, although fewer than those found in MTT-ON, due to the difference in throughput between both technologies. Other studies analyzing testis cDNA libraries from juvenile *P. monodon* acknowledged the existence of a high number of over-expressed transcripts in this tissue type [49]. This seems to be a common trait between cultured crustacean species [50,51,52] that share the same juvenile stage. Moreover, male-biased gene expression has been identified in juvenile stages of other arthropods such as insects [53,54].

### 3.5. Correlation of Gene Expression Levels between ONT and PacBio Samples

**Figure 5:**
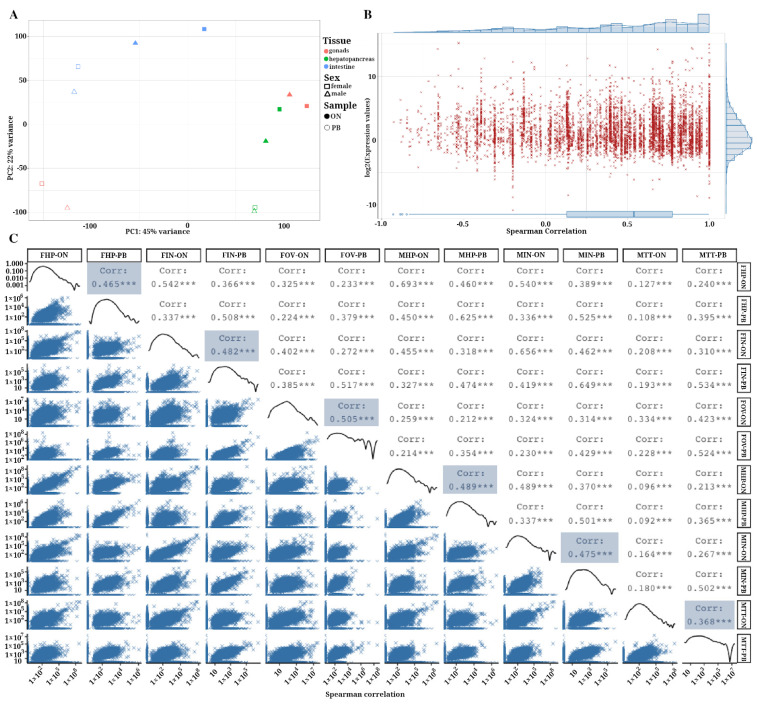
Correlation of gene expression between samples. (**A**) Principal component analysis showing the spatial relationship of each of the samples in the study. (**B**) Spearman correlation analysis using mean of normalized gene expression values in ONT and PacBio samples. The Y axis is showed in Log2 scale. (**C**) This correlogram shows level of correlation of normalized gene expression values using ONT and PacBio samples. For each sample, the Spearman correlation coefficient of the expression values was calculated (*** *p* < 0.01).

Due to differences in read length and throughput between both technologies, we decided to evaluate the correlation of gene expression values rather than transcript expression values between both technologies. To this end, levels of gene expression between the sequencing products of ONT and PacBio technologies were quantified using the DESeq2 normalized numbers of mapped cDNA reads, using filtered ONT reads and refined PacBio CCS reads (Supplementary Table S5). Refined CCS reads represent a highly accurate consensus of the sequencing product of 1 cDNA molecule using PacBio SMRT sequencing technology [55]. In the case of ONT, cDNA reads are linear fragments that correspond to the product of a single RNA molecule after reverse transcription and that are sequenced only once [6]. Thus, differences between the datasets should be due to the use of PCR products before the sequencing step in the PacBio sequencing pipeline and the obvious differences in sequencing throughput. Exploratory analysis of the samples using principal component analysis (PCA) of the gene expression profiles for the 12 samples showed the association between samples belonging to the same tissue (Figure 5A). However, samples from both technologies were distributed differently across the two components, indicating a strong technological bias, probably derived from the significant differences in sequencing depth. Correlation analysis using Spearman’s rank coefficients calculated using means of normalized gene expression values between both technologies by gene (Figure 5B) and by sample (tissue) (Figure 5C) showed positive correlation (<0.6) in both sets of analyses. The median of the Spearman’s rho coefficients when analyzing the average of normalized gene expression values, for both technologies, was 0.54 (Figure 5B). Gene-wise correlation levels between the two technologies ranged between 0.15 and 0.77, indicating that these values were independent of the degree of gene expression (Figure 5B). This implies that highly expressed genes are not better correlated, or vice versa.

The consistency of the expression values between the technologies by sample is shown in the correlogram in Figure 5C. The variable distribution along with the Spearman’s correlation values displayed on the right side of the plot indicate that, in general, there was a certain relationship between the number of normalized counts mapped when using ONT filtered reads and refined CCS reads when analyzed by sample. Spearman’s rho coefficients ranged from 0.368 in testis samples, the lowest correlation value, to 0.505 in ovary samples (*p* < 0.01). Levels of correlation were higher when comparing tissues from the same technology, as expected, showing in general a moderate positive correlation between their level of gene expression. Although outside the scope of this study, in which we are leveraging only long-read technologies, further investigations using technical replicates and Illumina RNA-seq sequencing should be done to cross compare gene expression levels against the standard method.

### 3.6. Comparison of Final Isoform Estimation and Functional Annotation

**Figure 6:**
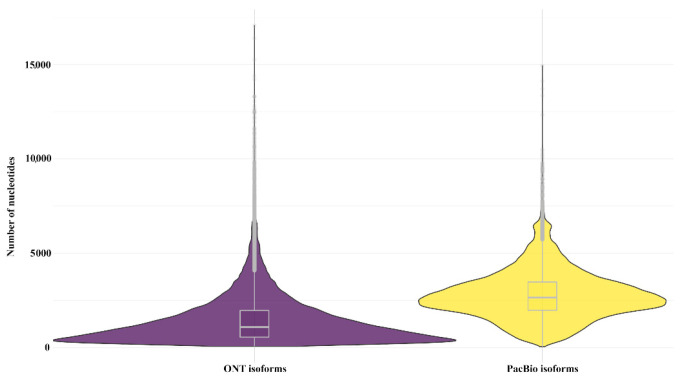
Size distribution of final isoforms obtained using ONT and PacBio sequencing technologies. The violet and yellow color for isoforms distribution of ONT and PacBio sequencing, respectively.

The final product of the PacBio IsoSeq3 pipeline consists of a set of final isoforms resulting from a clustering step for the refined CCS (or FLNC) reads. To obtain the most similar product between both technologies, we used FLAIR software v1.5.1 [35] to cluster and collapse the filtered sets of ONT reads into isoforms according to their chains of splice junctions. The total number of isoforms generated with the IsoSeq3 pipeline was 33,845. However, the total number of isoforms generated with the FLAIR workflow using transcripts from ONT samples rose to 112,878. Due to the considerable differences in the number of isoforms identified by the two technologies, we decided to further evaluate the completeness of both transcriptomes. To do this, we used the BUSCO pipeline [38] using the database of conserved arthropod genes, which contains 1013 BUSCO genes from 90 species. These analyses indicated that the *P. monodon* transcriptome obtained with ONT sequencing technology encoded for 71.3% BUSCO genes (Supplementary Table S6). Of these, 5.9% were complete single-copy and 65.4% were complete duplicated BUSCO genes. The percentage of fragmented BUSCOs was 9.7%, and 19% of BUSCOs from the arthropod database were missing from our ONT samples. In the case of PacBio technology, the isoforms obtained as final output of the IsoSeq3 pipeline represented 34.8% of complete BUSCOs; 28.0% of the complete BUSCOs identified corresponded to single copy and 6.8% corresponded to duplicated BUSCOs. The percentage of fragmented BUSCOs identified was 1.0%; therefore, 64.2% of the BUSCOs from the arthropod database was not represented in the PacBio transcriptome. As can be observed in Figure 6, the high number of fragmented BUSCOs in the case of the ONT final isoforms was expected due to the smaller size of the isoforms from the ONT samples, probably as a consequence of an elevated 3′ bias in our ONT transcripts. However, although ONT isoforms were clearly over-represented in our samples, the higher throughput obtained with ONT technology was crucial to achieve a higher level of completeness for the transcriptome in this study. It should also be noted that the level of completeness for these two transcriptomes, especially in the case of ONT samples, was not expected to be higher, as we only used samples from three tissues of juvenile *P. monodon*. Values above 98% completeness have been achieved in other studies with samples from nine tissues and from different developmental stages [56].

The six sets of isoforms obtained were functionally annotated using gene ontology (GO) terms. A WEGO 2.0 [57] plot showing the distribution of the GO term annotations (at hierarchical level 2) for different tissue types is depicted in Supplementary Figure S4. The number of isoforms from ON samples annotated using GO terms was higher than those annotated from PacBio samples (Supplementary Table S7). However, the number of isoforms identified, and the number of isoforms annotated using GO terms did not follow a linear relationship (Pearson’s correlation coefficient, PCC > 0.54) in the case of ONT samples, but for PacBio samples, the number of functional annotated isoforms showed a very high correlation (PCC > 0.99) with regard to the total number of isoforms obtained. Thus, and independently of the number of isoforms obtained for both technologies, profiles of annotated GO terms between both technologies were in general very similar when plotted using a logarithmic scale to account for the low abundance of some GO term categories (Supplementary Figure S4).

Annotation of final isoforms using KEGG orthology (KO) terms from the KEGG Automatic Annotation Server (KAAS) rendered a total of ~390 KEGG pathway categories. Similarly, the number of isoforms from ONT samples annotated using KO terms was higher than those annotated from PacBio samples in almost all cases, with the exception of sample MIN_ON. In this case, the number of isoforms annotated using KO terms followed a linear correlation in relation to the total number of isoforms in both technologies (ONT PCC ~0.8, PacBio PCC > 0.99), with this linear relationship higher in the case of PacBio samples, as in the previous analysis (Supplementary Table S8). In the same vein, the number of KO terms annotated for each KEGG category was highly correlated between technologies, with PCC > 0.83 (Supplementary Table S8). Although further exploration of the results obtained from the functional annotation of the final obtained isoforms was outside the scope of this study comparing the sequencing technologies, a relationship of the KO terms obtained for each sample is provided in Supplementary Table S9 and can be visualized using KEGG Mapper suite from KEGG (https://www.genome.jp/kegg/mapper.html, accessed on 1 August 2020).

## 4. Final Remarks

Here, we compared the main characteristics of the results obtained from the analysis of two transcriptomes of juvenile *Penaeus monodon* generated with the Oxford Nanopore Technologies (ONT) MinION platform and the Pacific Biosciences (PacBio) SEQUEL platform with IsoSeq3 workflow. The primary purpose of this work was to evaluate the bioinformatic workflows and combinations of software commonly used for transcriptomic analysis using long-read sequencing technologies alone. In the same vein, this work was motivated by the lack of benchmarking and comparative studies, mainly in non-model eukaryotes, leveraging both technologies with transcriptomic data. Thus, our results and analysis highlight the main differences between both technologies that, in light of the findings shown, should not be used indiscriminately in transcriptomic studies. While the PacBio IsoSeq3 protocol produces highly accurate consensus reads, the mappability of these reads to the reference genome was only slightly better than the mappability of ONT reads. Nevertheless, the higher accuracy values of PacBio reads obtained when aligned against the reference genome could be crucial in performing rigorous analyses such as sensitive identification of novel junctions and isoforms [33]. However, the ONT platform produced libraries with smaller mean read sizes and higher error rates than PacBio. Still, our data indicate that the substantially higher sequencing throughput obtained with ONT MinION sequencing compared to PacBio sequencing makes ONT more suitable for leveraging quantitative analysis and discovering novel transcripts. It should be noted that the smaller sizes of ONT reads obtained (and therefore the final isoforms) could be due to the known 3′ bias toward the end of the transcripts [6,7,42] (possibly derived from 3′ bias of first strand cDNA synthesis step). The 3′ bias was also found in direct-RNA sequencing of ONT as well [1,58]. This bias could be due to fragmentation during the library preparation process (mainly when using cDNA reads) or as a result from technical limitations such as pore blocking [6]. One consequence of the 3′ bias is that the quantification of transcripts is yet another challenge to overcome when using cDNA reads from ONT [42]. Our study, has no technical replicates, which is a limitation. However, RNA samples were aliquots from the same pool for ONT and PacBio sequencing, enabling a fair technical comparison.

Differences observed in sequencing depth between ONT and PacBio technologies could likewise cause the lack of correlation between gene expression levels found for both technologies. An investigation of optimal sequencing depth for gene/isoform expression level quantification using long-read sequencing technologies would be required to meaningfully compare both technologies in this regard. Indeed, it is still a common practice to use short reads to improve abundance estimation of transcripts and genes produced by long-read sequencing technologies [42,59]. We also identified other limitations not directly related to the sequencing technology but to applying and scaling existing tools to larger genomes. The rapid development of these technologies has produced a large number of new tools and software that should be comprehensively evaluated to allow the scientific community to identify and choose adequately between them. Some open-source resources and repositories for long-read software and tools are available on GitHub (https://github.com/B-UMMI/long-read-catalog, accessed on 1 August 2020) and on the long-read-tools.org database [4].

In conclusion, although both ONT and PacBio technologies can overcome the main limitations of using short-read technologies for transcriptome sequencing, they still present a number of challenges that need to be addressed to provide a definitive solution that marks the end of short-read sequencing in the transcriptomic area [60]. Our study highlights the high sequencing throughput achieved using ONT, as one of the strengths of this technology over PacBio. However, one of the greatest advantages of the IsoSeq approach is the possibility of leveraging a standardized pipeline that provides a final output of high-quality isoforms. Thus, each of these technologies have their own strengths and weakness and should therefore not be used interchangeably in transcriptomic studies.

## Figures and Tables

**Table 1:** Overview of the samples and summary of the sequencing output obtained from 12 libraries using ONT and PacBio sequencing technologies. Sample abbreviations showed in this results: female-hepatopancreas (FHP), male-hepatopancreas (MHP), female-intestine (FIN), male-intestine (MHP), ovary (FOV), and testis (MTT); samples sequenced using Pacbio (PB) and samples sequenced using ONT (ON).

Sample ID	Platform	Sex	Tissue	Reads, Total Bases (Gb)	Read Number	Average Read Length (b)	Read Length N50
FHP_PB	PacBio	Female	Hepatopancreas	3562	1,550,060	2297	2487
FIN_PB	PacBio	Female	Intestine	4980	1,967,020	2531	2962
FOV_PB	PacBio	Female	Ovary	4553	1,589,542	2864	3752
MHP_PB	PacBio	Male	Hepatopancreas	5199	2,701,407	1924	2367
MIN_PB	PacBio	Male	Intestine	4221	1,949,961	2164	2738
MTT_PB	PacBio	Male	Testis	4787	2,081,856	2299	3023
FHP_ON	ONT	Female	Hepatopancreas	12,465	10,556,858	1181	1681
FIN_ON	ONT	Female	Intestine	5973	5,944,869	1005	1230
FOV_ON	ONT	Female	Ovary	14,093	8,359,907	1686	2613
MHP_ON	ONT	Male	Hepatopancreas	8178	6,570,975	1245	1845
MIN_ON	ONT	Male	Intestine	5541	5,344,621	1037	1325
MTT_ON	ONT	Male	Testis	10,919	9,223,658	1184	1746

(Gb, gigabases; b, bases).

**Table 2:** Summary of output obtained after using two error-correction algorithms on ONT filtered reads.

	Sample ID	Read Number	Average of Reads Length	Mapped Bases (%)/Clipped (%)	Mapped Reads (%)	Unmapped Reads	Supplementary Alignments	General Error Rate
ONT filtered	FHP_ON	7,794,835	1147	99.57/96.51	99.48	40,170	273,464	0.12
	FIN_ON	5,237,429	996	99.01/93.18	98.84	60,905	290,981	0.12
	FOV_ON	5,641,133	1719	98.73/90.59	97.68	130,620	596,976	0.12
	MHP_ON	4,925,279	1222	99.61/96.90	99.46	26,745	143,951	0.12
	MIN_ON	3,883,617	1029	99.25/93.62	99.16	32,594	185,252	0.12
	MTT_ON	6,720,316	1185	98.78/95.00	98.24	118,541	288,694	0.12
TranscriptClean	FHP_ON	7,794,835	1174	99.58/96.59	99.48	40,170	273,464	0.02
	FIN_ON	5,237,429	1016	99.03/93.31	98.84	60,905	275,315	0.02
	FOV_ON	5,641,133	1728	98.74/90.64	97.68	130,620	596,976	0.02
	MHP_ON	4,925,279	1247	99.62/96.97	99.46	26,745	143,951	0.01
	MIN_ON	3,883,617	1050	99.26/93.75	99.16	32,594	185,252	0.02
	MTT_ON	6,720,316	1207	98.80/95.09	98.24	118,541	288,694	0.02
MECAT	FHP_ON	712,368	1395	97.10/92.63	96.5	24,736	114,199	0.06
	FIN_ON	715,312	1282	96.42/91.45	95.64	31,215	125,218	0.06
	FOV_ON	1,701,918	1747	97.37/92.31	96.2	64,661	242,980	0.05
	MHP_ON	438,541	1321	96.94/92.41	96.06	17,286	60,668	0.06
	MIN_ON	427,321	1327	96.29/91.49	95.64	18,613	71,757	0.06
	MTT_ON	1,033,153	1346	97.34/92.82	96.73	33,799	163,571	0.07

**Table 3:** Measurements of accuracy, sensitivity (S), and precision (P) calculated as in Burset and Guigó (1996) [48] for all samples. For more information on these measurements: https://ccb.jhu.edu/software/StringTie/gffcompare.shtml (accessed on 1 August 2020).

	Base Level				Intron Chain Level				Transcript Level			
	Reference File		Merged File		Reference File		Merged File		Reference File		Merged File	
	S	P	S	P	S	P	S	P	S	P	S	P
FHP_ON	100	57.7	99.9	96.7	100	60.1	100	99.7	99.5	59.7	100	96.8
FIN_ON	100	57.7	99.9	96.7	100	60.1	100	99.7	99.5	59.6	100	96.7
FOV_ON	100	57.7	99.9	96.7	100	60.1	100	99.7	99.5	59.7	100	96.8
MHP_ON	100	57.7	99.9	96.7	100	60.1	100	99.7	99.5	59.6	100	96.7
MIN_ON	100	57.7	99.9	96.7	100	60.1	100	99.7	99.5	59.7	100	96.7
MTT_ON	100	57.7	99.9	96.8	100	60.1	100	99.7	99.5	59.6	100	96.7
FHP_ONt	100	57.7	99.9	96.7	100	60.1	100	99.7	99.5	59.7	100	96.8
FIN_ONt	100	57.7	99.9	96.7	100	60.1	100	99.7	99.5	59.6	100	96.7
FOV_ONt	100	57.7	99.9	96.7	100	60.1	100	99.7	99.5	59.7	100	96.8
MHP_ONt	100	57.7	99.9	96.7	100	60.1	100	99.7	99.5	59.6	100	96.7
MIN_ONt	100	57.7	99.9	96.7	100	60.1	100	99.7	99.5	59.7	100	96.7
MTT_ONt	100	57.7	99.9	96.8	100	60.1	100	99.7	99.5	59.6	100	96.7
FHP_PB	100	57.7	99.9	96.8	100	60.1	100	99.7	99.5	59.7	100	96.8
FIN_PB	100	57.7	99.9	96.8	100	60.1	100	99.7	99.5	59.7	100	96.8
FOV_PB	100	57.7	99.9	96.8	100	60.1	100	99.7	99.5	59.7	100	96.8
MHP_PB	100	57.7	99.9	96.8	100	60.1	100	99.7	99.5	59.7	100	96.8
MIN_PB	100	57.7	99.9	96.8	100	60.1	100	99.7	99.5	59.7	100	96.8
MTT_PB	100	57.7	99.9	96.8	100	60.1	100	99.7	99.5	59.7	100	96.8

## Data Availability

Sequencing libraries from this study have been deposited at the National Center for Biotechnology Information Sequence Read Archive (Bioproject: PRJNA749966). Final isoforms obtained in this study are available as the Supplementary Material.

## Supplementary Materials

The following are available online at https://www.mdpi.com/article/10.3390/life11080862/s1. Table S1. Quality values from *Penaeus monodon* RNA samples. Table S2. Sequencing summary and detailed statistics of the filtered ONT reads and refined CCS reads used in the analysis. Table S3. Detailed statistics of the alignments of the filtered ONT reads and refined CCS reads against the *P. monodon* genome. Table S4. GFFCompare results from the comparison of the transcripts assembled by StringTie2 to the annotated transcripts of the original reference file, represented also in Figure 4. Table S5. DESeq2-normalized gene expression values for the 12 libraries in the study. Table S6. Detailed results from the analysis of the final isoforms using BUSCO pipeline. Table S7. Summary of functional annotated isoforms using WEGO 2.0. Pearson correlation coefficient was obtained using “correl” function in each paired dataset. Table S8. Summary of functional annotated isoforms using KAAS. Pearson correlation coefficient was obtained using “correl” function in each paired dataset. Table S9. Detailed annotation of isoforms using KO terms. Figure S1. (A) Summary of the sequencing throughput obtained using for ONT (reads) and PacBio (subreads) samples. (B) Violin plots representing the distribution of the base 10 logarithmic transformation of the read length obtained after sequencing for each sample from each technology. Figure S2. Bivariate plot of aligned read length against read percent identity and marginal histograms. ONT samples shown in blue; PacBio samples shown in red. Plots generated with Nanopack package [22]; Figure S3. Analysis of transcripts obtained after alignment using StringTie2 and compared with the reference file using filtered and trimmed ONT reads and high quality clustered reads from IsoSeq3 pipeline in case of PacBio samples. Annotation codes correspond to transcript classification codes obtained using the transcript file output from StringTie2 for each sample and the reference file and compared using GffCompare [33]. Figure S4. Distribution of the GO term annotations at hierarchical level 2 in each tissue using WEGO 2.0 [57]. Supplementary Data: Set of final isoforms obtained using FLAIR for ONT samples and from IseoSeq3 pipeline in case of PacBio samples.
