# Supplementary figures and images for: Comparative Analysis of PacBio and Oxford Nanopore Sequencing Technologies for Transcriptomic Landscape Identification of Penaeus monodon
Source: Life (Basel). 2021 Aug 23;11(8):862. doi: 10.3390/life11080862 (PMC8399832; doi:10.3390/life11080862)

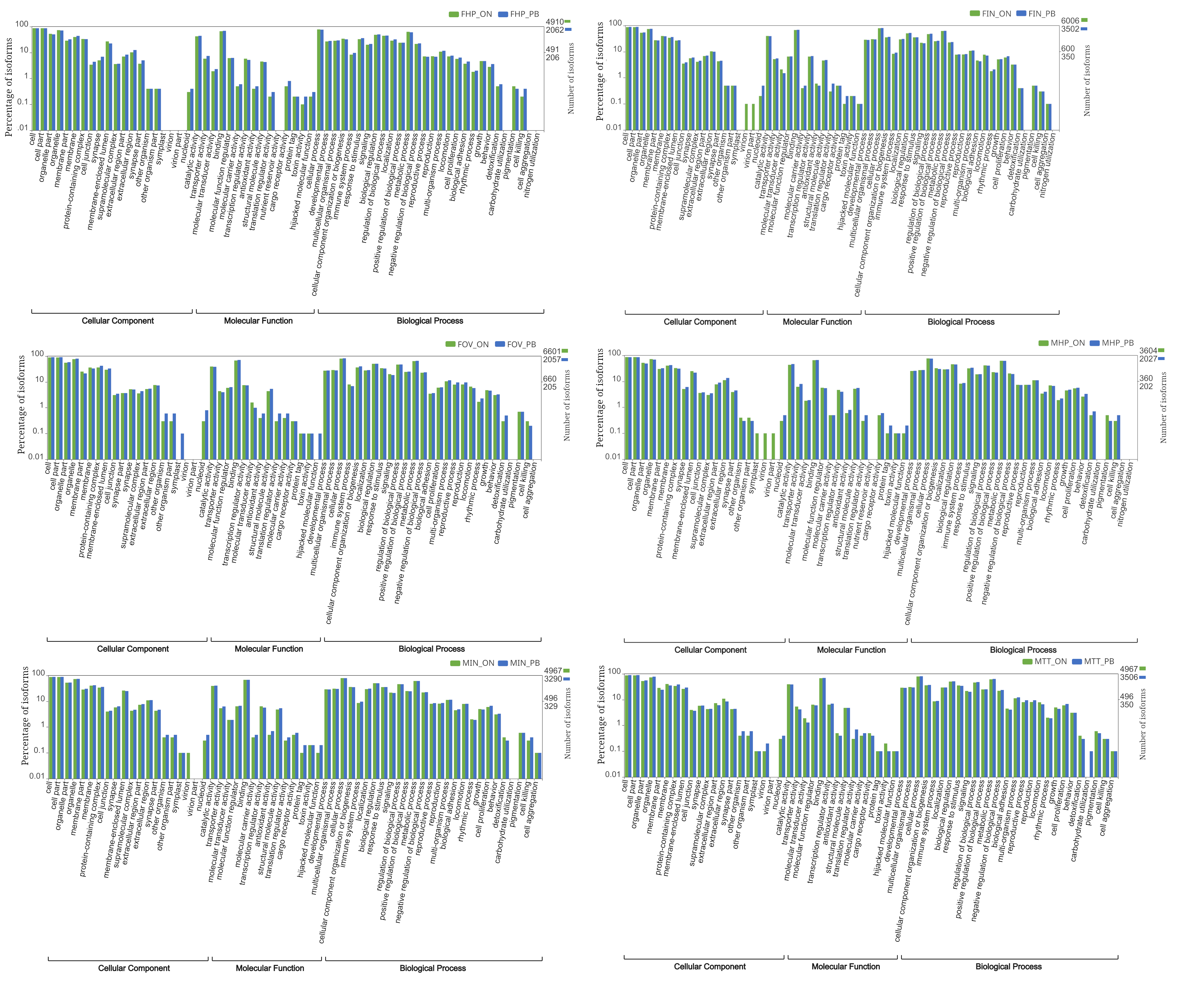

Supplement: Supplementary file 1 [file life-11-00862-s001.zip › Suppl_Material/suppl_Figure_4.tif]

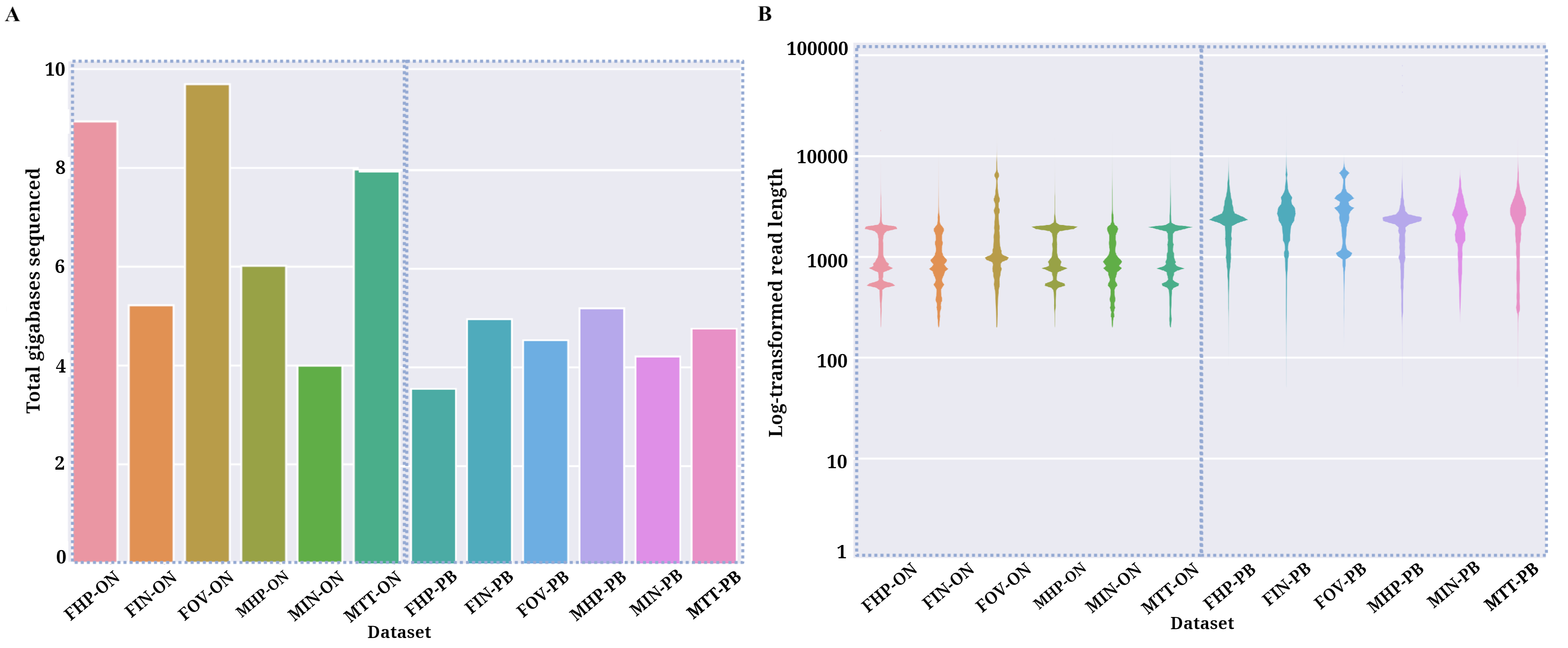

Supplement: Supplementary file 1 [file life-11-00862-s001.zip › Suppl_Material/Suppl_Figure_1.tif]

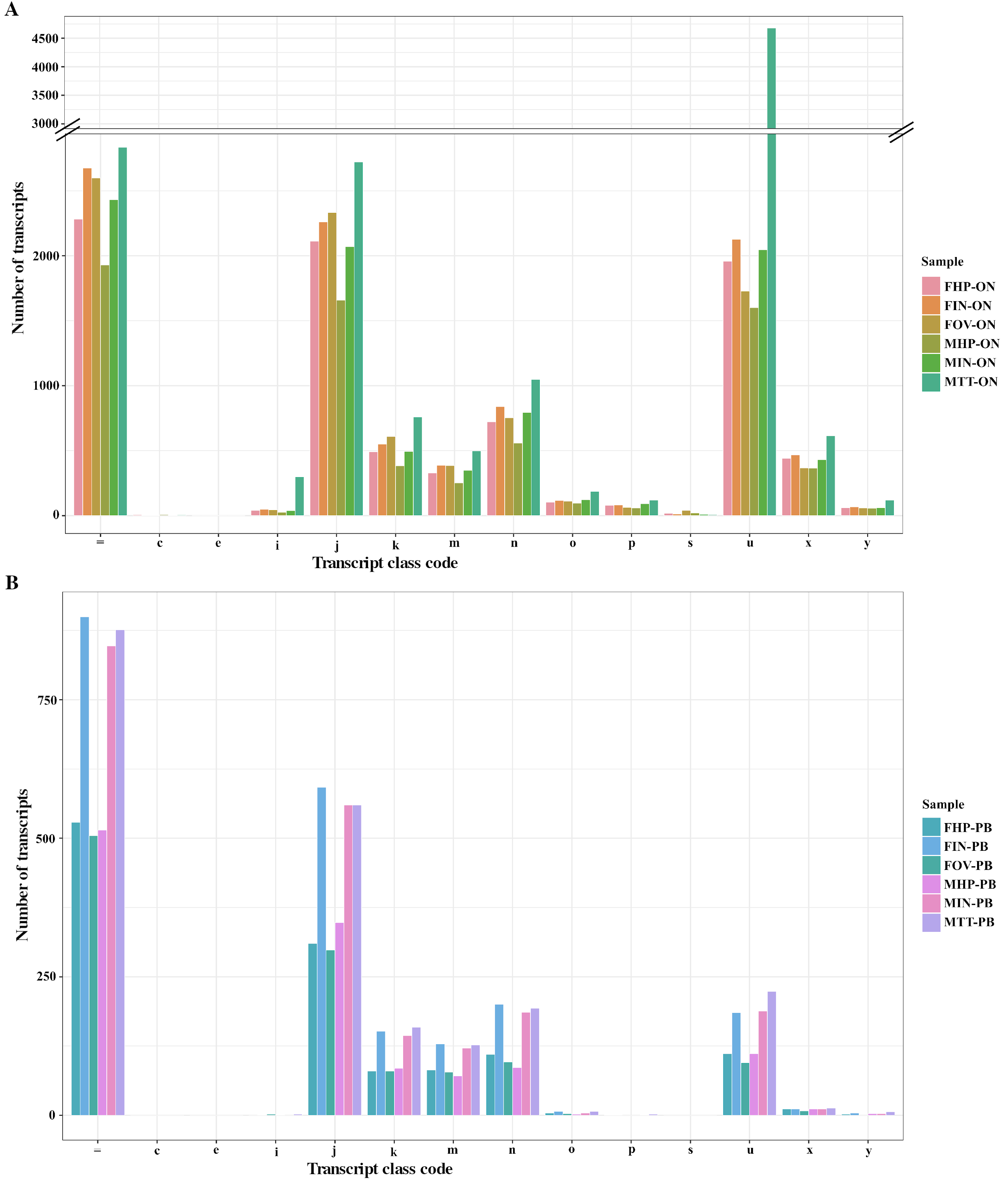

Supplement: Supplementary file 1 [file life-11-00862-s001.zip › Suppl_Material/Suppl_Figure_3.tif]

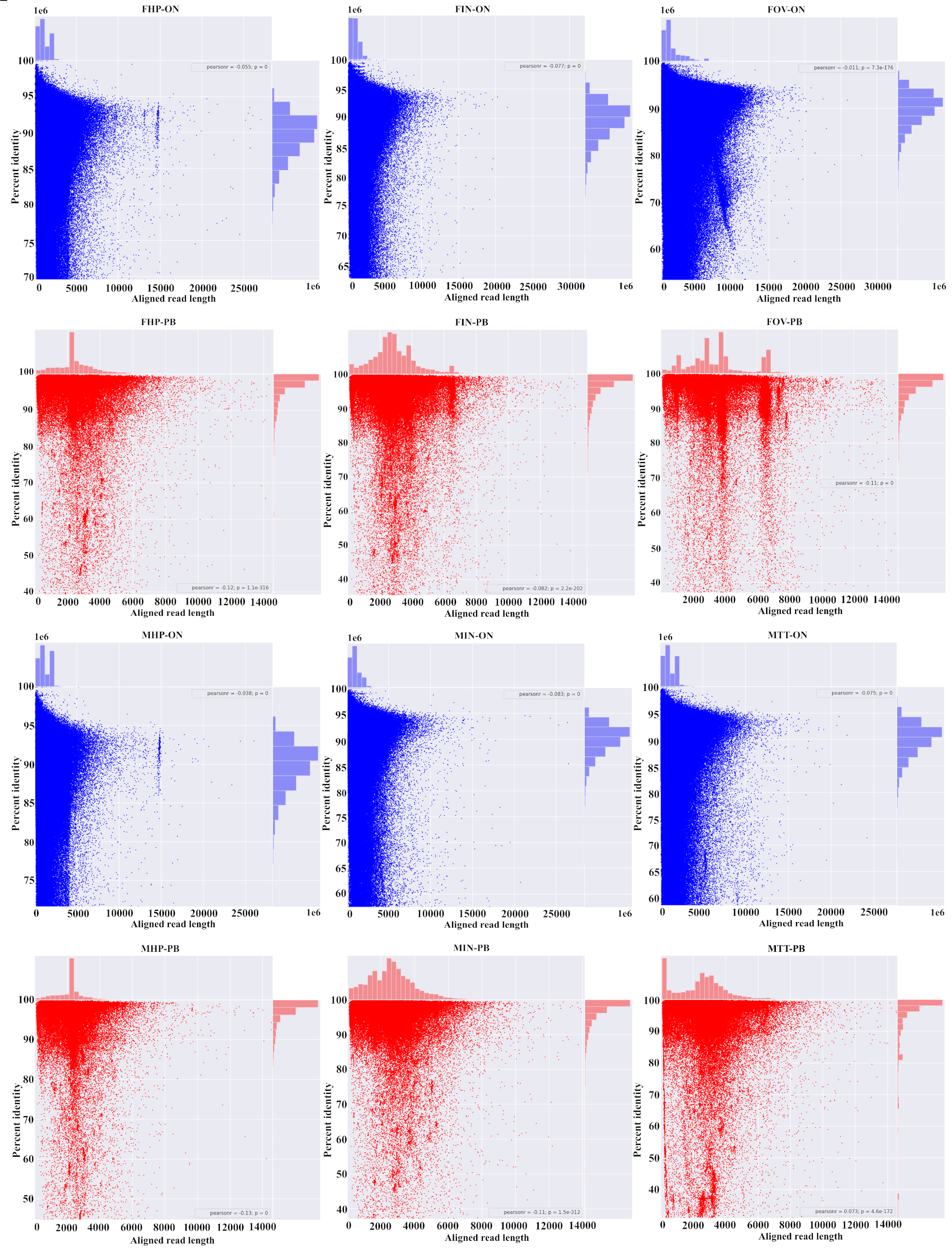

Supplement: Supplementary file 1 [file life-11-00862-s001.zip › Suppl_Material/Suppl_Figure_2.tiff]
